# A Rare Case of Mucosa-Associated Lymphoid Tissue Lymphoma in the Ileum

**DOI:** 10.7759/cureus.32851

**Published:** 2022-12-22

**Authors:** Nitesh Badwaik, Pankaj Gharde, Yashwant Lamture, Shailja Singh, Rushikesh Shukla

**Affiliations:** 1 General Surgery, Jawaharlal Nehru Medical College, Datta Meghe Institute of Medical Sciences, Wardha, IND

**Keywords:** h. pylori infection, and cd21+, cd35+, cd20+, igm+, igd, marginal zone b cells, mucosa-associated lymphoid tissue (malt) lymphoma

## Abstract

The body contains mucosa-associated lymphoid tissue (MALT), with the greatest amount located in the gastrointestinal (Gl) tract. Lymphoma may form when the cell growth in this tissue is aberrant. The small intestine is a common extranodular site of lymphoma, a systemic illness. Additionally, it has been proposed that MALT lymphomas (MALTomas) arise as a result of chronic and persistent immunological activation, either of an autoimmune or infectious type. The MALToma that develops in the duodenum is typically thought to be unrelated to *Helicobacter pylori* infection. However, some examples show that lymphoma regressed when *H. pylori* were removed.

## Introduction

Mucosa-associated lymphoid tissue (MALT) lymphoma was described first by Isaacson (&) Wright in 1983. They found that the peripheral lymph nodes and MALT shared more histological characteristics with primary low-grade gastric B cell lymphomas and immunoproliferative intestinal illness. The MALTomas can develop at a variety of extranodal sites, such as the stomach (70%), the lung (14%), the ocular adnexa (12%), the thyroid (4%), and the small intestine [1]. Mucosa-associated lymphoid tissue is an extranodal marginal zone B-cell lymphoma [2]. It is the cancerous multiplication of B cells of lymphoid tissue in the marginal area [3]. Morphologically it is heterogeneous small B cells which include marginal cells. The cells resemble small lymphocytes, centroblast-like cells, scattered immunoblasts, and monocytoid cells. Also, they include invaded lymphoma cells throughout the epithelium with the multiplication of plasma cells in the lamina propria of the mucosa [4]. The MALToma symptoms differ based on the site where the organs are affected because it is a localized condition. In fewer than 5% of instances, it has non-specific symptoms, including malaise, weight loss, low-grade fever, and abdominal pain [5].

## Case presentation

**Figure 1:**
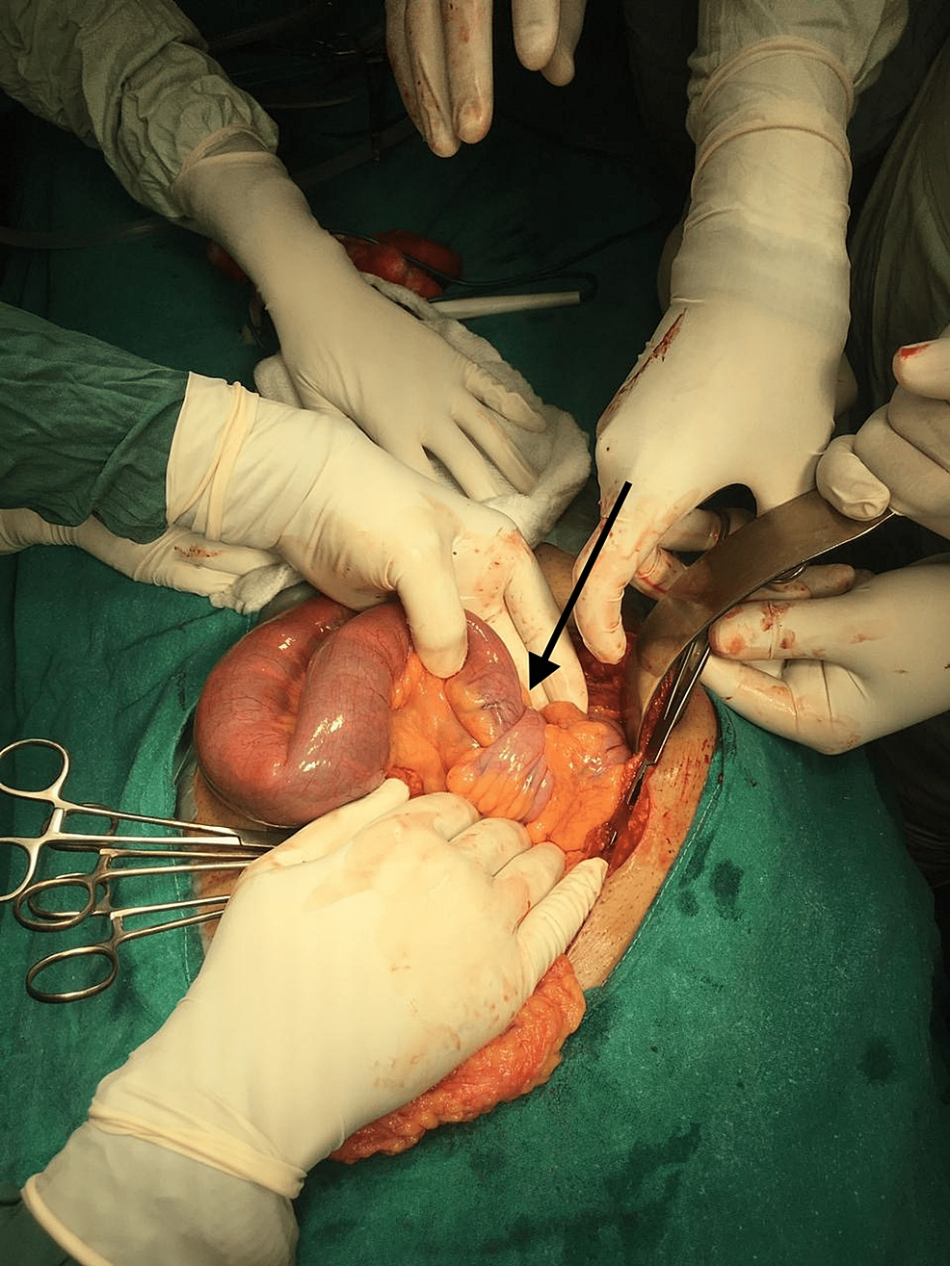
Intraoperative picture immediately post the opening up of the abdomen The black arrow shows intussusception with the classical telescopic appearance of the ileal segment.

**Figure 2:**
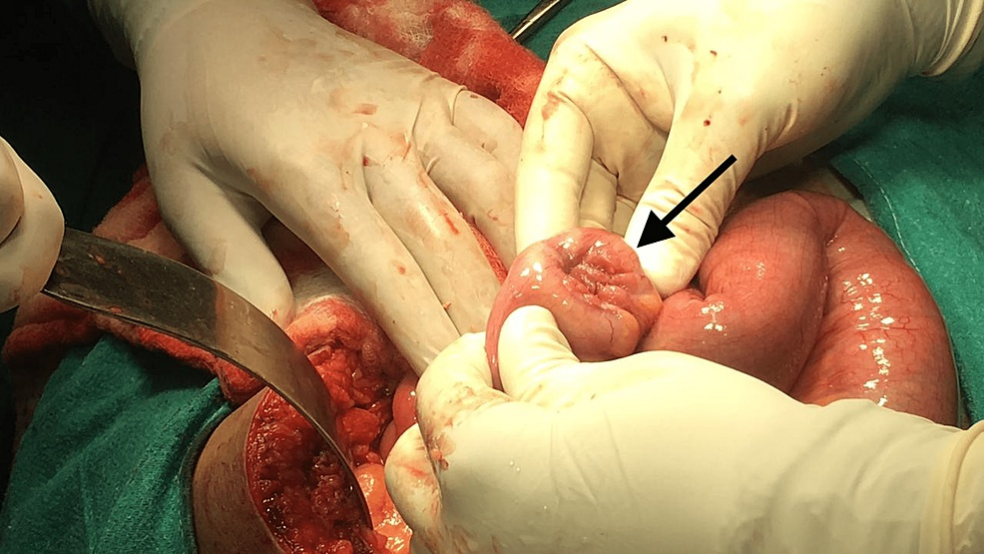
Nodular growth noted over the ileal segment The black arrow shows the site of mucosa-associated lymphoid tissue (MALT) acting as a lead point for intussusception.

A 38-year-old male patient developed sudden onset of pain in the abdomen, which was colicky in nature, non-radiating, moderate to severe, gradually progressive in intensity, and associated with one episode of non-bilious non-projectile vomiting with food and water content. He had not passed stools for the past two days. There was no history of fever, cough, or burning micturition. There was no significant past medical or surgical history. He occasionally drank about 60ml of alcohol once a month. The general physical and systemic examinations were normal except for tenderness in the right iliac fossa. Contrast-enhanced computed tomography (CECT) abdomen and pelvis was done which was suggestive of telescopic appearance in the ileal segment intussusception. The patient underwent resection and anastomosis of the ileal segment (Figures 1, 2).

**Figure 3:**
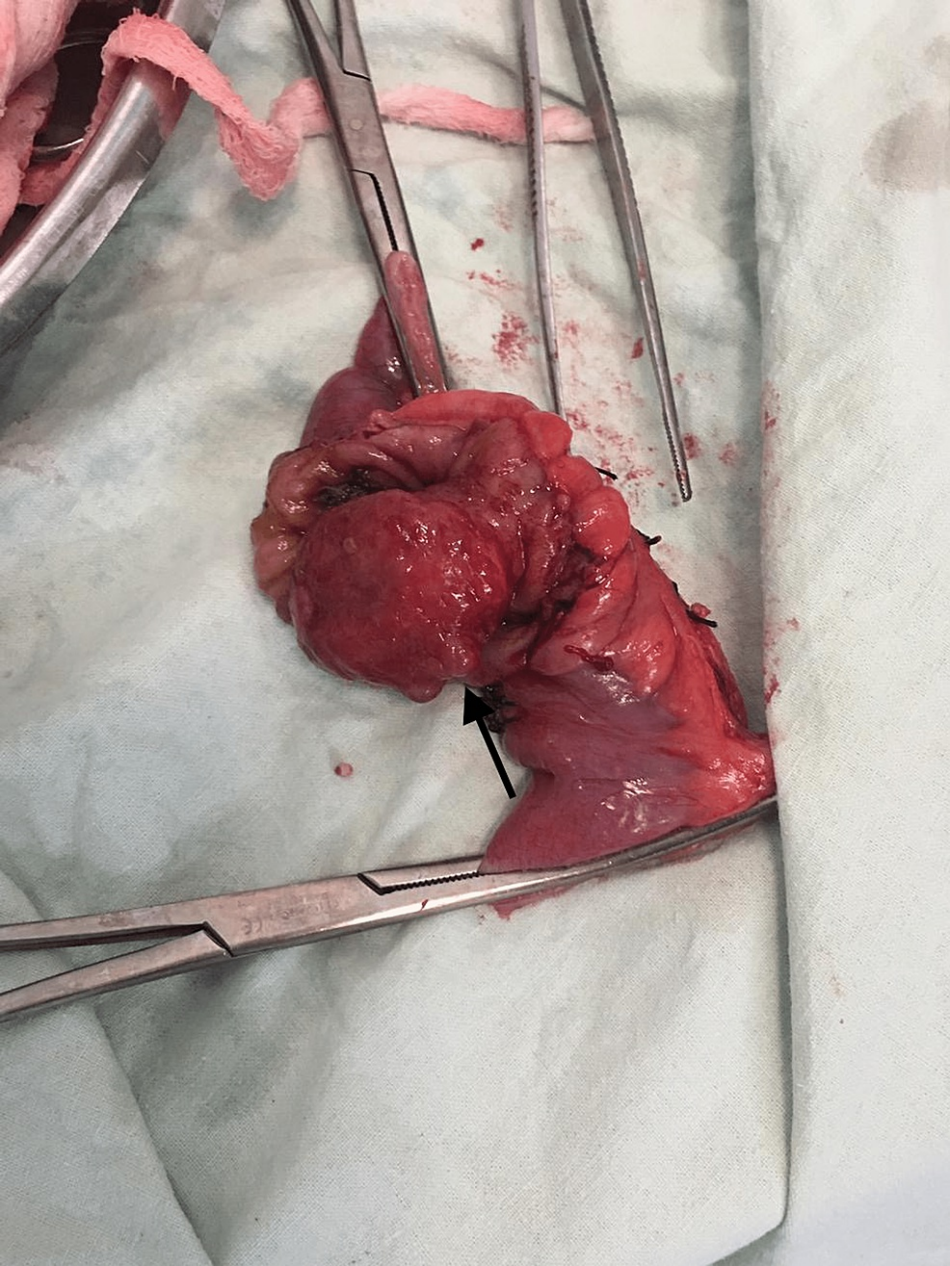
Postoperative photo of the resected specimen

Postoperatively the specimen was examined and the gross appearance of the resected ileal segment was suggestive of a growth over which intussusception was present (Figure 3).

**Figure 4:**
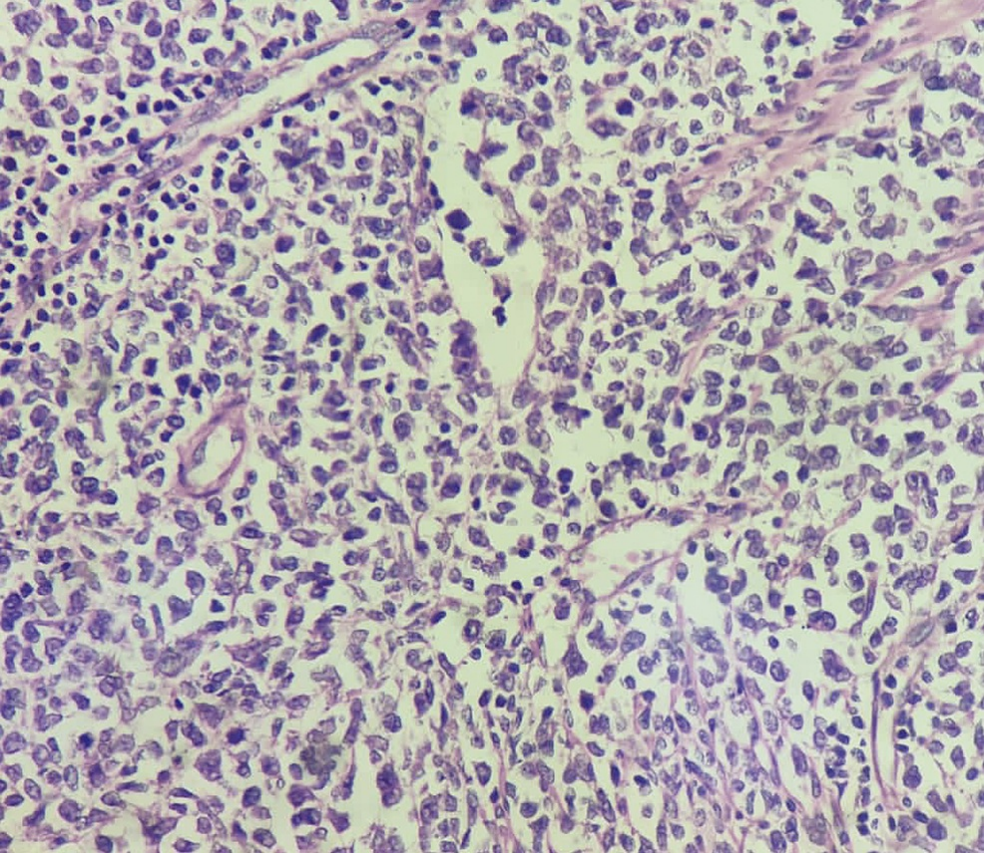
Non-Hodgkins lymphoma diffuse pattern MALT type MALToma seen at 40x magnification; hematoxylin and eosin stain MALT: Mucosa-associated lymphoid tissue, MALToma: MALT lymphoma

**Figure 5:**
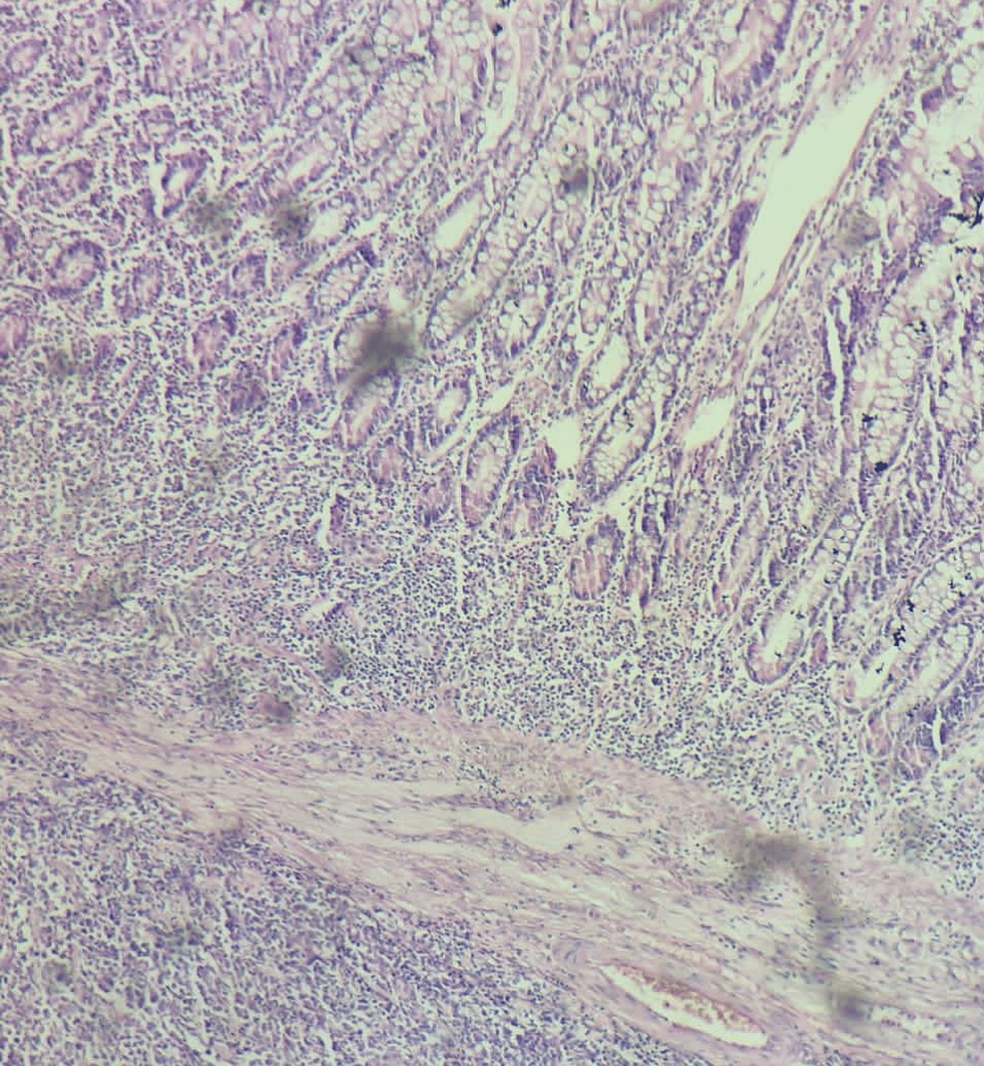
Slide showing non-Hodgkins lymphoma with MALT-like lymphoma at 10x magnification; hematoxylin and eosin stain MALT: Mucosa-associated lymphoid tissue

 The specimen was sent for histopathological examination and was suggestive of non-Hodgkin's lymphoma (Figures 4, 5).

The patient's postoperative period was uneventful, and he was discharged with a follow-up date six months post-surgery.

## Discussion

The most amount of MALT is located in the GI tract. Lymphoma may form when the cell growth in this tissue is aberrant. A MALToma of the GI tract makes up 50% of cases that are present. The small intestine, the stomach, and the colon are the next most frequent sites where a MALToma can occur. Small intestine lymphomas specifically account for up to 40% of primary GI lymphomas and 25% of primary malignancies at this level. The small intestine is a common extra-nodular site of lymphoma, which is a systemic illness [6]. The ileum and cecum in the intestine are the most affected areas, likely as a result of the plentiful lymphoid tissue there. Multiple localizations are also seen in 5% to 15% of cases [7]. Strong correlations have been found between *Helicobacter pylori *infection and gastric MALTomas. This is the most prevalent infectious agent connected to worldwide cancers during an *H. pylori* infection. Three chromosomal translocations, namely t(11;18) (q21;921), t(1;14)(p22;q32), and t(14;18)(q32;q21), help turn healthy B cells into cancerous clones. Nuclear factor kappa B (NF-B), which is implicated in immunity, inflammatory processes, and apoptosis, is stimulated as a result of these translocations [8]. Additionally, it has been proposed that MALTomas arise as a result of chronic and persistent immunological activation, either of an autoimmune or infectious type [9]. A primary GI lymphoma must meet five requirements outlined by Dawson: (1) the lack of peripheral lymphadenopathy at the time of presentation, (2) the absence of enlarged mediastinal lymph nodes, (3) a normal total and differential white blood cell count, (4) the predominance of a bowel lesion at the time of laparotomy with only nearby lymph nodes clearly affected, and (5) the absence of lymphomatous involvement of the liver and spleen [10]. The invasion of the marginal zone and the diffuse dissemination of the tumor into the surrounding tissue are the histopathological characteristics of MALToma. The MALToma cells exhibit the same cytological and immunophenotypic characteristics as marginal zone B cells (immunoglobulin (Ig)D, IgM+, cluster of differentiation (CD)20+, CD35+, and CD21+) [1]. Multiple tiny erosions, nodular elevations, and diffuse erythema in the duodenal bulb are the symptoms of duodenal MALTomas. According to specific descriptions, the symptoms consist of several active ulcer craters without stomach involvement, each having nodular and erythematous edges. Since the MALToma is a primarily gastrointestinal lymphoma that develops in the duodenum, it is typically thought to be unrelated to *H. pylori* infection. However, some examples show that lymphoma regressed when *H. pylori* were removed.

An unusual discovery in our case is the MALToma in the ileum. According to early descriptions, the endoscopic pattern consists of many reddish or white masses with a polished and smooth mucosa look. Five patterns were distinguished in the first classification of ileal lymphoma endoscopic features: mucosal fold thickening alone, nodular pattern, infiltrating pattern, ulcerative pattern, and mosaic pattern [7]. Even though it is rare, a possible complication is when a proximal section of the intestine telescopes into the lumen of the neighboring section during intussusception. Intussusception in adults is incredibly uncommon and typically only occurs in younger populations. Adult symptoms can be very different. As nonspecific symptoms of adult intussusception, bleeding, nausea, changes in bowel habits, vomiting, and constipation with abdominal swelling have been noted [2]. The development of capsule endoscopy and the double balloon technique of push-and-pull enteroscopy has improved the diagnosis of small intestinal lymphomas. Ileal MALToma is anecdotic, and only a handful of occurrences have been reported in the literature. The majority of cases show a lack of *H. pylori* infection, leaving the cause of this condition unexplained. Nearly half of the patients present with a single tumor that resembles a carcinoma in physical appearance; multiple masses or lymphomatoid polyposis are less common [7]. By performing a biopsy on the afflicted area along with morphologic, immunophenotypic, and genetic studies on the biopsy specimen, a reliable diagnosis of MALToma can be obtained [11]. For the detection of MALTomas, positron emission tomography-computed tomography (PET-CT) is more effective. In one study, 42% of early MALTomas were found using PET-CT [12].

The most sensitive procedures are endoscopic submucosal resection or ultrasound-guided endoscopic fine-needle aspiration biopsy [3]. Nongastric MALToma treatment after diagnosis is identical to that for other non-Hodgkin lymphomas. A thorough physical workup, a laboratory assessment that includes a full blood count with differentials, an all-inclusive metabolic panel, and a quantification of lactate dehydrogenase as well as a CECT of the pelvis, chest, and abdomen, should all be included in the workup [2]. Nearly 90% of patients with stomach MALToma survive for 10 years, and about 70% remain disease-free [1].

## Conclusions

The third most prevalent non-lymphoma Hodgkin's subtype is MALToma. Since MALToma of the ileum is uncommon, treating *H. pylori*, which is a major factor in the formation of MALToma, should be the first line of treatment. It is difficult to make a quick clinical diagnosis because the diagnosis is only made intraoperatively for intestinal blockage. The incorporation of both chemotherapy and surgery is thought to be superior to other approaches. In the case described above, the ileal segment was resected and anastomosis was done. The incidence rate as well as the proper diagnostic and treatment procedure for intestinal MALToma is not standardized in previous studies and literature, which proves to be a major problem. 
